# Early catheter ablation vs. antiarrhythmic drugs in treatment-naïve ischaemic ventricular tachyarrhythmias: a meta-analysis of randomized controlled trials

**DOI:** 10.1093/europace/euag073

**Published:** 2026-06-26

**Authors:** Tiago Moura Façanha, Anna Isabelle Gomes Pereira, Maria Fernanda Carvalho Garcia, Enzo Tunala Mendonça, João Vitor Jacobsen Ramos, Leonardo Antunes Mesquita

**Affiliations:** Faculty of Medicine, University of Fortaleza, Fortaleza, Ce, Brazil; Faculty of Medicine, FAMETRO University Center, Manaus, AM, Brazil; Faculty of Medical and Health Sciences, SUPREMA, Juiz de Fora, Brazil; Faculty of Medicine, University of São Paulo, São Paulo, SP, Brazil; Faculty of Medicine, Santa Casa de Misericórdia de Vitória, Vitória, ES, Brazil; Department of Electrophysiology, Hospital Madre Teresa, Avenida Raja Gabaglia 1002, Gutierrez, Belo Horizonte, MG 30441-070, Brazil

**Keywords:** Ventricular tachyarrhythmias, Treatment-naïve, Drug-naïve, First-line therapy, Catheter ablation, Drug therapy

## Abstract

**Introduction:**

Antiarrhythmic drugs (AADs) have traditionally been used as first-line therapy for ventricular tachycardia (VT) but are frequently associated with systemic toxicities and drug interactions. Therefore, we performed a meta-analysis comparing early catheter ablation (CA) with AAD therapy as initial treatment for VT in patients with prior myocardial infarction.

**Methods and results:**

PubMed, Embase, and the Cochrane Library were searched for randomized controlled trials comparing CA vs. AAD in patients naïve to both strategies. Meta-analysis was performed using Review Manager. Three randomized trials including 618 patients were analysed. No significant differences were observed in all-cause mortality, appropriate implantable cardioverter-defibrillator (ICD) therapies (including shocks and antitachycardia pacing), ventricular arrhythmia storm, hospitalizations, or adverse events between strategies. Leave-one-out sensitivity analysis identified one trial as the primary contributor to between-study heterogeneity.

**Conclusion:**

Early catheter ablation provides similar efficacy to first-line antiarrhythmic drug therapy for major arrhythmic and survival outcomes, with reductions in hospitalizations and treatment-related adverse events, although largely driven by one trial. These findings support consideration of CA as a first-line therapeutic strategy in selected patients with ischaemic VT.

## Graphical Abstract

**Graphical Abstract:**
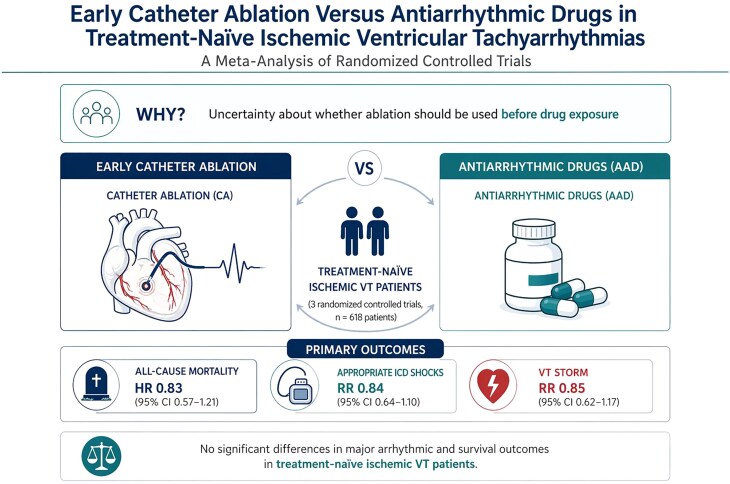

## Introduction

Implantable cardioverter-defibrillators (ICDs) represent the cornerstone strategy for the prevention of sudden cardiac death in patients with ventricular tachycardia (VT).^1–3^ However, they do not prevent VT recurrence, and ICD therapies are associated with impaired quality of life, frequent hospitalizations, and progression of heart failure (HF).^2,4,5^ Antiarrhythmic drug (AAD) therapy, particularly amiodarone and sotalol, has traditionally been the first-line strategy to suppress ventricular arrhythmias in this population,^1,6^ although its efficacy is limited and frequently associated with systemic toxicities, drug interactions, and long-term safety concerns.^6,7^ In this context, catheter ablation (CA) has emerged as an effective strategy for reducing VT recurrences and ICD therapies, particularly in patients with drug-refractory arrhythmias, and technological advances have improved procedural success and safety.^1,2,8,9^

Most prior randomized trials evaluated ablation as a second-line therapy after AAD failure, leaving uncertainty about whether ablation should be used before drug exposure.^1,8^ Recurrent VT during this period may contribute to clinical deterioration, repeated ICD therapies, and progressive electrical instability, potentially affecting long-term outcomes.^2,5^

An updated meta-analysis suggested that early CA reduces VT recurrence and ICD therapies; however, substantial heterogeneity in intervention timing and background antiarrhythmic therapy, as well as inclusion of previously treated patients, precluded definitive conclusions regarding its role as first-line therapy in treatment-naïve populations.^10^ More recently, randomized trials have compared early CA with initial AAD therapy in patients with ischaemic cardiomyopathy and VT naïve to both strategies, although their findings have been heterogeneous and underpowered for definitive conclusions.^2,8,10^

Therefore, we conducted a systematic review and meta-analysis of randomized controlled trials (RCTs) to evaluate whether early CA, compared with initial AAD therapy, has an effect on arrhythmic and clinical outcomes in patients with prior myocardial infarction and VTs naïve to both treatments. We aimed to clarify the impact of early ablation on mortality, arrhythmia recurrence, ICD therapies, hospitalizations, and adverse events, thereby addressing a critical gap in the management strategy for ischaemic VT.

## Methods

This systematic review and meta-analysis was performed and reported in accordance with the Cochrane Collaboration Handbook for Systematic Review of Interventions^11^ and the Preferred Reporting Items for Systematic Reviews and Meta-Analysis (PRISMA) Statement guidelines^12^

### Eligibility criteria

In this systematic review and meta-analysis, only studies following predefined eligibility criteria were included: (i) RCTs, (ii) studies comparing CA to AAD therapy as first-line treatment, and (iii) studies enrolling patients with prior myocardial infarction and sustained ventricular tachycardia or ventricular fibrillation (VT/VF).

No minimum follow-up was required, and studies were excluded if they (i) were non-randomized, (ii) included patients with previous CA or AAD exposure, or (iii) enrolled patients with contraindication to all protocol-allowed AAD options.

### Search strategy

We systematically searched PubMed, Embase, and the Cochrane Library with the following search terms: ‘ventricular arrhythmias’, ‘ventricular fibrillation’, ‘treatment-naïve’, ‘ablation’, and ‘drug therapy’. The complete search strategy is provided in the Supplementary material online, *Appendix*.

The reference lists of all included studies, previous systematic reviews, and meta-analyses were also searched manually for any additional studies. Two authors independently extracted the data following predefined search criteria and quality assessment. The prospective meta-analysis protocol was registered in the International Prospective Register of Systematic Reviews (PROSPERO) on 20 January 2026, under ID CRD420261287375.

### Endpoints

The primary outcomes included all-cause mortality, appropriate ICD shocks, and ventricular storms at any time. Secondary outcomes included appropriate ICD therapies and hospitalizations. Non-fatal adverse events were considered safety outcomes. Definitions of ventricular tachycardia and related arrhythmic outcomes varied across the included trials; therefore, detailed outcome definitions were extracted and are summarized in Supplementary material online, Table S1.

### Quality assessment

We evaluated the risk of bias in randomized studies using the Cochrane Risk of Bias 2 (RoB 2) tool (Supplementary material online, Figure S1).^13^ Two authors completed the risk of bias assessment. Disagreements were resolved through a consensus after discussing reasons for discrepancy.

### Statistical analysis

Treatment effects were pooled using a DerSimonian–Laird random-effects model with 95% confidence intervals (CIs). Statistical heterogeneity was assessed using Cochran’s Q test and quantified with the I^2^ statistic. *P*-values of <0.10 and I^2^ values of 25%, 50%, and 75% were considered indicative of low, moderate, and high heterogeneity, respectively. Given the small number of trials included, pooled estimates were interpreted cautiously. Leave-one-out sensitivity analyses were performed to evaluate the influence of individual studies on pooled effect estimates and between-study heterogeneity. Statistical analyses were conducted using Review Manager version 5.4 (Cochrane Collaboration, Copenhagen, Denmark).^14^

Effect measures were selected according to the original reporting of each trial. All-cause mortality was analysed as a time-to-event outcome and pooled as hazard ratios (HRs) using the generic inverse-variance method. A supplementary analysis using risk ratios (RRs) was also performed ( Supplementary material online, Figure S1). Secondary endpoints were synthesized as risk ratios based on the number of patients experiencing at least one event, since time-to-event estimates were not consistently available across studies.

## Results

### Study selection and characteristics

**Figure 1:**
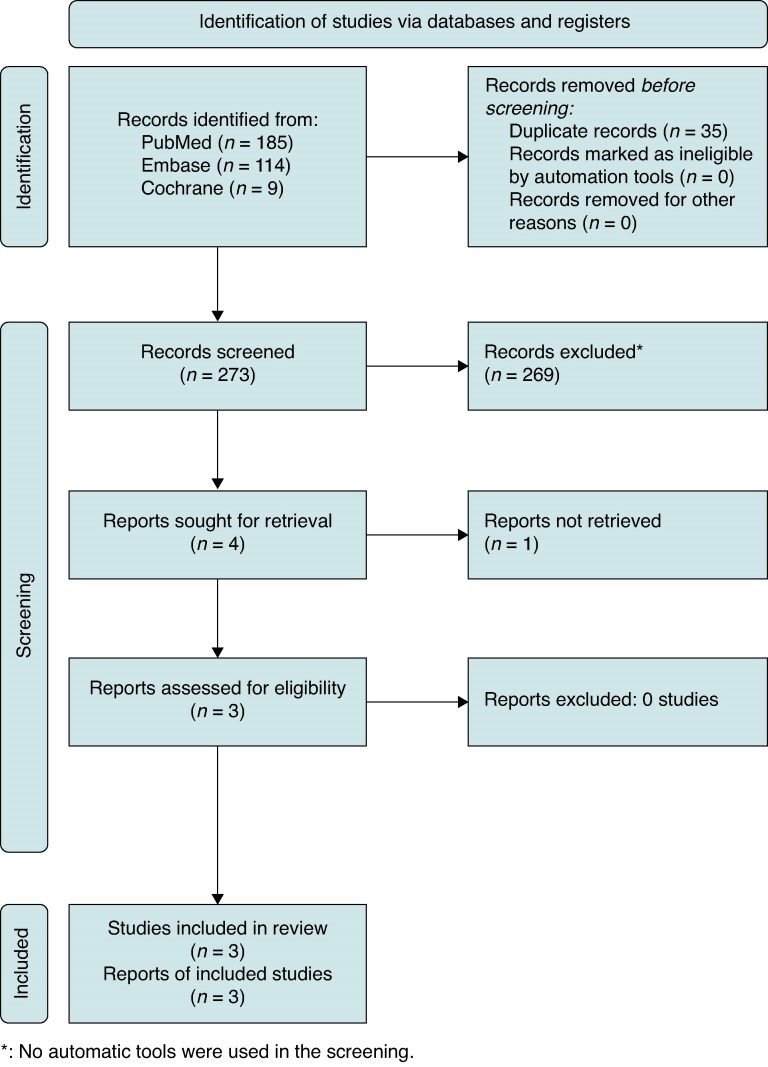
PRISMA flow diagram of study screening and selection.

The initial search yielded 308 records. After removal of 35 duplicate records, 273 studies remained for screening. Following title and abstract review, 269 were excluded. Four reports were sought for retrieval, of which one could not be retrieved. Ultimately, three RCTs met inclusion criteria and were included in the final analysis, comprising a total of 618 patients (*Figure 1*).

Across the three RCTs, 302 patients (48.9%) underwent CA and 316 (51.1%) received AAD therapy. All studies enrolled patients with VT in the setting of prior ischaemic cardiomyopathy. Study characteristics are summarized in *Table 1*. Across trials, CA consisted predominantly of substrate-guided radiofrequency ablation supported by electroanatomic mapping systems, with programmed ventricular stimulation performed when feasible to assess inducibility. In the medical therapy arm, amiodarone was used as the primary antiarrhythmic agent across all trials,^15–17^ while sotalol was allowed as an alternative in selected patients. In SURVIVE-VT,^16^ antiarrhythmic therapy consisted of amiodarone or sotalol, administered either alone or in combination with carvedilol.

**Table 1:** Baseline characteristics of included studies

	Sapp *et al.*, 2025^15^ (*n* = 416)		Arenal *et al.*, 2022^16^ (*n* = 144)		Raatikainen *et al.*, 2025^17^ (*n* = 58)	
Characteristics	CA (*n* = 203)	AAD (*n* = 213)	CA (*n* = 71)	AAD (*n* = 73)	CA (*n* = 28)	AAD (*n* = 30)
Follow-up, months^a^	51.6 (30.0–68.4)		23.8 (16.6–24.0)	23.3 (9.4–23.9)	24.0	
Age, years	67.7 ± 8.6	68.4 ± 8.0	70 (63–75)	71 (64–76)	65.9 ± 8.2	67.1 ± 7.3
Male sex, *n* (%)	193 (95.1)	197 (92.5)	70 (98.6)	68 (93.2)	26 (93)	28 (93)
BMI (kg/m^2^)	NR	NR	27.3 (25.2–31.6)	27.6 (25.9–30.0)	28.8 ± 4.5	28.5 ± 3.6
DM, *n* (%)	79 (38.9)	83 (39.0)	21 (29.6)	15 (20.5)	9 (32)	9 (30)
HTN, *n* (%)	160 (78.8)	169 (79.3)	56 (78.9)	47 (64.4)	19 (68)	17 (57)
LVEF, %	34.0 ± 11.0	34.3 ± 10.3	35 (26–41)	33 (25–40)	36 ± 11	37 ± 13
RI, *n* (%)	31 (15.3)	23 (10.8)	8 (11.3)	7 (9.6)	NR	NR
Creatinine (mg/dL)	NR	NR	1.05 (0.87–1.28)	1.02 (0.88–1.15)	NR	NR
Previous CABG, *n* (%)	82 (40.4)	88 (41.3)	18 (26.5)	12 (17.1)	NR	NR
Previous PCI, *n* (%)	128 (63.1)	121 (56.8)	26 (38.2)	26 (37.1)	NR	NR
Last or first MI, years	13.3 ± 9.9	14.8 ± 10.4	14 (6–24)	14 (7–23)	13.4 ± 8.0	14.5 ± 8.9
NYHA I, *n* (%)	87 (42.9)	89 (41.8)	31 (44.3)	31 (42.5)	NR	NR
NYHA II, *n* (%)	99 (48.8)	107 (50.2)	33 (47.1)	37 (50.7)	NR	NR
NYHA III, *n* (%)	17 (8.4)	17 (8.0)	6 (8.6)	5 (6.8)	NR	NR
AF or flutter, *n* (%)	70 (34.5)	72 (33.8)	9 (13.6)	8 (12.3)	6 (21)	9 (30)
Prior ICD, *n* (%)	179 (88.2)	188 (88.3)	68 (95.8)^b^	70 (95.9)^b^	28 (100)^b^	30 (100)^b^
CRT, *n* (%)	43 (21.2)	36 (16.9)	11 (15.5)	13 (18.1)	3 (11)	4 (13)
ICD1, *n* (%)	66 (32.5)	77 (36.2)	55 (77.5)	54 (75.0)	9 (32.0)	11 (37.0)
ICD2, *n* (%)	94 (46.3)	100 (46.9)	5 (7.0)	5 (6.9)	17 (61.0)	15 (59.0)
BB use, *n* (%)	NR	NR	69 (97.2)	62 (86.1)	28 (100)	29 (97)
ACEI/ARB use, *n* (%)	NR	NR	70 (98.6)	65 (90.3)	26 (93)	27 (90)
Anterior MI, *n* (%)	NR	NR	25 (35.2)	31 (42.5)	13 (46)	7 (23)
Inferior MI, *n* (%)	NR	NR	46 (64.8)	40 (54.8)	9 (32)	16 (53)
Lateral MI, *n* (%)	NR	NR	6 (8.5)	12 (16.4)	0 (0)	2 (6.7)

Values are presented as mean ± standard deviation, median (interquartile range), or number (%) according to the original study report.

AAD, antiarrhythmic drug; ACEI, angiotensin-converting enzyme inhibitor; AF, atrial fibrillation; ARB, angiotensin receptor blocker; BB, beta-blocker; BMI, body mass index; CA, catheter ablation; CABG, coronary artery bypass grafting; DM, diabetes mellitus; HTN, hypertension; ICD, implantable cardioverter-defibrillator; ICD1, implantable cardioverter-defibrillator single chamber; ICD2, implantable cardioverter-defibrillator dual chamber; CRT, cardiac resynchronization therapy; LVEF, left ventricular ejection fraction; MI, myocardial infarction; NYHA, New York Heart Association; NR, not reported; PCI, percutaneous coronary intervention; RI, renal insufficiency.

^a^Average follow-up, mean, or median.

^b^By inclusion criteria.

The pooled cohort included predominantly male patients (>90%) with VT secondary to ischaemic cardiomyopathy, with a mean age in the mid-60s to early 70s. Hypertension was the most frequent comorbidity.

### Pooled analysis of all studies

**Figure 2:**
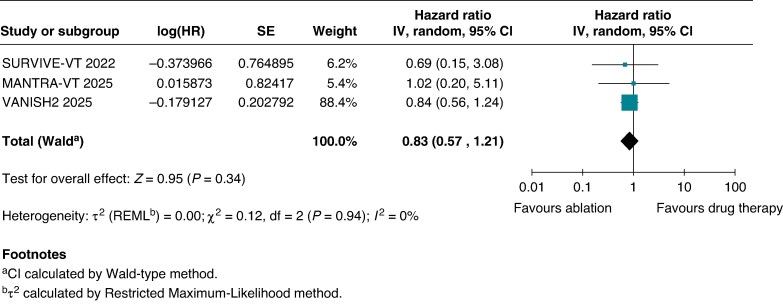
Early catheter ablation vs. first-line antiarrhythmic drug therapy for all-cause mortality.

**Figure 3:**
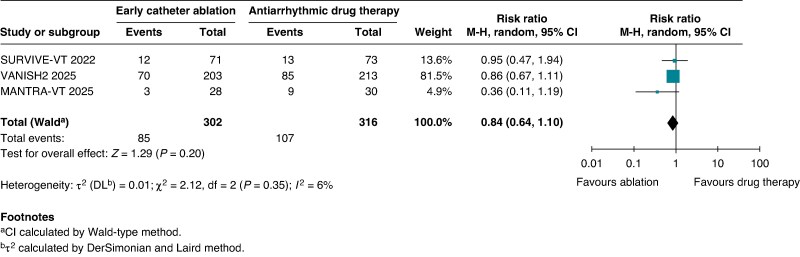
Early catheter ablation vs. first-line antiarrhythmic drug therapy for appropriate ICD shocks.

**Figure 4:**
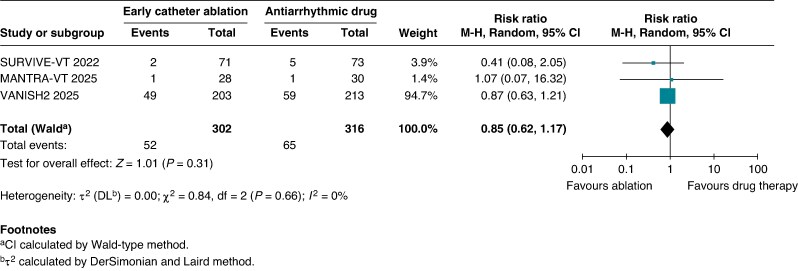
Early catheter ablation vs. first-line antiarrhythmic drug therapy for ventricular storm.

**Figure 5:**
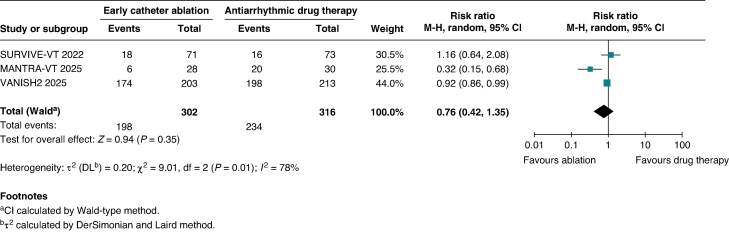
Early catheter ablation vs. first-line antiarrhythmic drug therapy for appropriate ICD therapies.

**Figure 6:**
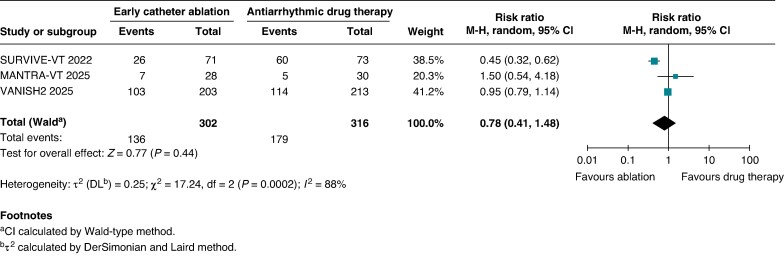
Early catheter ablation vs. first-line antiarrhythmic drug therapy for hospitalizations.

**Figure 7:**
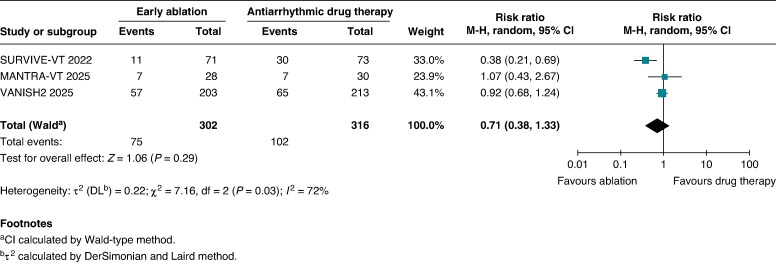
Early catheter ablation vs. first-line antiarrhythmic drug therapy for adverse events.

In patients undergoing early CA, compared with first-line AAD therapy, there was no significant reduction in all-cause mortality (16.9% vs. 19.3%; HR 0.83; 95% CI 0.57–1.21; *P* = 0.34; I^2^ = 0%; *Figure 2*). Similarly, no significant differences were observed in appropriate ICD shocks (28.1% vs. 33.9%; RR 0.84; 95% CI 0.64–1.10; *P* = 0.20; I^2^ = 6%; *Figure 3*) or ventricular storm (17.2% vs. 20.6%; RR 0.85; 95% CI 0.62–1.17; *P* = 0.31; I^2^ = 0%; *Figure 4*). Rates of appropriate ICD therapies (65.5% vs. 74.1%; RR 0.76; 95% CI 0.42–1.35; *P* = 0.35; I^2^ = 78%; *Figure 5*) were also comparable between groups. However, early CA was associated with numerically lower rates in hospitalizations (45.0% vs. 56.6%; RR 0.78; 95% CI 0.41–1.48; *P* = 0.44; I^2^ = 88%; *Figure 6*) and adverse events (24.8% vs. 32.3%; RR 0.71; 95% CI 0.38–1.33; *P* = 0.29; I^2^ = 72%; *Figure 7*), although these differences did not reach statistical significance.

### Sensitivity analysis

To further assess the robustness of the findings and explore sources of heterogeneity, leave-one-out sensitivity analyses were performed for hospitalizations and adverse events. Exclusion of MANTRA-VT or VANISH2 did not meaningfully modify the pooled estimates or heterogeneity. However, removal of SURVIVE-VT eliminated heterogeneity entirely (I^2^ = 0%) for both outcomes.

Following exclusion of SURVIVE-VT, CA remained directionally associated with lower risk, although statistical significance was lost for adverse events (27.7% vs. 29.6%; RR 0.93; 95% CI 0.70–1.24; *P* = 0.64; I^2^ = 0%; Supplementary material online, Figure S2) and hospitalizations (47.6% vs. 48.9%; RR 0.96; 95% CI 0.80–1.15; *P* = 0.67; I^2^ = 0%; Supplementary material online, Figure S3).

These findings suggest that SURVIVE-VT substantially contributed to between-study heterogeneity and that the apparent numerical reduction in hospitalizations and adverse events is largely attributable to this trial. The sensitivity analysis forest plots are provided in the Supplementary material online, *Appendix*.

### Quality assessment

The RoB 2 tool was used to assess methodological quality. For all-cause mortality, no study was judged to be at high risk of bias (see Supplementary material online, *Table S2*). For adverse events, however, all trials were rated as having high overall risk of bias, primarily driven by concerns in the domain of selection of the reported result. Notably, most studies were considered at low risk in other domains, including the randomization process, missing outcome data, and measurement of the outcome. These considerations were considered when interpreting pooled estimates, particularly for adverse events. The risk of bias summary is provided in the Supplementary material online, *Appendix*.

## Discussion

This meta-analysis of randomized trials provides a contemporary synthesis comparing early CA with first-line AAD therapy in treatment-naïve patients with ischaemic VT. Overall, early ablation was not associated with reductions in mortality, major arrhythmic endpoints, hospitalizations, or adverse events.

### Mortality and arrhythmic outcomes

Across pooled analyses, early ablation did not reduce all-cause mortality, appropriate ICD shocks, overall appropriate ICD therapies, or ventricular storm. These findings are consistent with prior randomized data showing that although CA reduces arrhythmia burden, this has not translated into a survival advantage. Given the multifactorial determinants of mortality in ischaemic cardiomyopathy—particularly progressive HF and non-arrhythmic causes—the absence of a mortality signal is not unexpected. Differences in follow-up duration across trials may also have influenced outcome ascertainment, particularly for mortality and recurrent arrhythmic events, which could require longer observation periods to detect meaningful differences. The neutral effect on arrhythmic endpoints may reflect limited statistical power, heterogeneity in ICD programming and endpoint definitions, and differences in ablation strategies across trials. Moreover, the dynamic and progressive scar substrate inherent to ischaemic cardiomyopathy may attenuate the effect of early intervention on established re-entrant circuits. These data suggest that early ablation should not be interpreted as a mortality-modifying intervention, but rather as a strategy aimed at improving clinical stability and treatment tolerance.

### Hospitalizations and adverse events

Early ablation was associated with numerically lower rates of hospitalizations and adverse events, although these differences were not statistically significant. The observed reductions were largely driven by the SURVIVE-VT trial, which reported a higher burden of drug-related complications and hospital admissions in the AAD arm. Variations in patient selection, endpoint definitions, and event adjudication likely contributed to the heterogeneity observed across studies.

A plausible explanation is that chronic AAD therapy exposes patients to cumulative toxicity, intolerance, and drug–drug interactions, frequently resulting in hospitalizations for dose adjustments, adverse effects, or recurrent arrhythmias. In contrast, CA represents a time-limited intervention that may reduce ongoing pharmacologic exposure and its associated morbidity, rather than directly altering arrhythmic risk.

### Reframing risk: ablation vs. drug-related harm

Catheter ablation for VT has traditionally been reserved for patients who fail or are intolerant to AAD therapy, largely due to concerns regarding procedural risk. However, the present findings invite reconsideration of this sequencing strategy. Although ablation is often perceived as the higher-risk intervention, adverse events appeared numerically more frequent in patients treated with AADs, underscoring that the cumulative risks of chronic pharmacologic therapy may be underrecognized.

These observations suggest a broader reframing of treatment-related risk. Beyond procedural complications, clinicians should also consider the long-term morbidity associated with sustained AAD exposure, particularly in older patients with substantial comorbidity burden.

### Comparison with previous literature

Our findings are consistent with prior randomized trials and meta-analyses^18–20^ showing that CA reduces recurrent ventricular arrhythmias and appropriate ICD therapies in ischaemic VT, without a clear impact on all-cause mortality.

Unlike most previous studies,^18,20^ which predominantly included patients failing AAD therapy, our analysis focused on treatment-naïve individuals, directly comparing first-line ablation with initial pharmacologic therapy. The neutral mortality effect likely reflects the multifactorial nature of outcomes in ischaemic cardiomyopathy, whereas the observed lower rates in adverse events and hospitalizations appear largely related to avoidance of drug-related toxicity rather than a direct survival.^19,20^

### Clinical implications and paradigm shift

Taken together, these findings support reconsideration of the traditional stepwise approach in which CA is reserved for failure of AAD therapy. Although early ablation was not associated with improved survival or complete suppression of arrhythmic events, it achieved comparable major clinical outcomes and showed a numerical trend towards lower treatment-related morbidity and healthcare utilization.

From a practical standpoint, these data suggest that early ablation may be reasonably discussed as an upfront strategy in selected patients, particularly when long-term drug tolerance, comorbidity burden, or patient preference favour avoidance of chronic pharmacologic therapy. This does not imply replacement of AADs in all cases; rather, it supports a more individualized treatment algorithm in which ablation is considered a legitimate first-line option alongside pharmacologic therapy.

### Limitations

This meta-analysis included a limited number of randomized trials and a modest total sample size, reducing statistical power for infrequent outcomes, such as mortality. Some endpoints—particularly hospitalizations and adverse events—were predominantly influenced by individual studies, which may impact the stability of pooled estimates. Differences in study design, ablation strategy, background medical therapy, ICD programming, and endpoint definitions likely contributed to observed heterogeneity. Furthermore, the use of the DerSimonian–Laird random-effects model with only three trials may underestimate statistical uncertainty, particularly for outcomes with substantial heterogeneity; accordingly, these pooled estimates should be interpreted conservatively. Variability in follow-up duration also limits definitive conclusions regarding long-term comparative effectiveness.

## Conclusions

In patients with ischaemic VT naïve to both treatment strategies, early CA appears to provide clinical outcomes comparable to first-line AAD therapy, without increasing mortality or arrhythmic risk. Although numerically lower rates of treatment-related morbidity were observed, these findings were influenced by individual trials and should be interpreted cautiously. Overall, these data support consideration of early ablation as a reasonable first-line strategy in carefully selected patients.

## Supplementary Material

euag073_Supplementary_Data

## Data Availability

The data supporting the findings of this study are available from the corresponding author upon reasonable request.

## Protocol registration

This systematic review and meta-analysis was registered in PROSPERO (CRD420261287375).
