# Supplementary material for: Early catheter ablation vs. antiarrhythmic drugs in treatment-naïve ischaemic ventricular tachyarrhythmias: a meta-analysis of randomized controlled trials
Source: Europace. 2026 Jun 26;28(6):euag073. doi: 10.1093/europace/euag073 (PMC13303077; doi:10.1093/europace/euag073)
Supplement: euag073_Supplementary_Data [file euag073_supplementary_data.docx]

**Supplementary Materials**

This supplementary appendix provides additional methodological details, definitions of outcome variables and supporting tables and figures related to the analyses presented in the main manuscript. It includes the search strategy used in PubMed, Embase and Cochrane Library, definition of ventricular tachycardia used in each study, risk-of-bias assessment performed using the RoB 2 tool, risk ratio analysis for all-cause mortality and leave-one-out sensitivity analysis performed using Review Manager.

**Full search strategy:**("ventricular tachyarrhythmias" OR  “Ventricular dysrhythmias” OR “Malignant ventricular arrhythmias” OR “Ventricular arrhythmias” OR "ventricular tachycardia" OR "ventricular fibrillation") AND (naive OR "treatment-naïve" OR "drug-naïve" OR "therapy-naïve" OR unexposed OR "never treated" OR "no prior treatment" OR "no previous therapy" OR "no prior antiarrhythmic" OR "antiarrhythmic-naive" OR “ablation naive” OR “no prior ablation” OR "first-line therapy" OR "first-line strategy") AND (ICD OR RFCA OR ablation OR "catheter ablation" OR "radiofrequency ablation" OR "substrate ablation" OR "scar homogenization") AND ("drug therapy" OR amiodarone OR AAD OR sotalol OR Mexiletine OR Dronedarone)

**Supplementary Table 1.** *Ventricular tachycardia definitions.*

| STUDY | DEFINITION |
| --- | --- |
| MANTRA-VT 2025 | At least two documented episodes of symptomatic VT/VF and the last one within the preceding 12 months. |
| VANISH2 2025 | Ventricular tachycardia events: sustained monomorphic ventricular tachycardia terminated by pharmacologic therapy or electrical cardioversion; three or more episodes, including one symptomatic episode of ventricular tachycardia treated with antitachycardia pacing by an ICD; five or more episodes of monomorphic ventricular tachycardia regardless of symptoms; one or more appropriate ICD shocks; or three episodes of sustained ventricular tachycardia within 24 hours. |
| SURVIVE-VT 2022 | Very symptomatic VT defined as: 1) sustained VT treated using ICD shock (<6 months); and 2) sustained VTs with syncope, even if terminated with antitachycardia pacing. |

**Table S1.** Standardized definitions of ventricular tachycardia used across the studies included in the meta-analysis.

**Supplementary Table 2.** *Risk-of-bias summary for randomized studies (RoB 2) for the adverse events outcome*

| ***Study*** | ***Bias from randomization process*** | ***Bias due to deviations from intended interventions*** | ***Bias due to missing outcome data*** | ***Bias in measurement of the outcomes*** | ***Bias in selection of the reported result*** | ***Overall risk of bias*** |
| --- | --- | --- | --- | --- | --- | --- |
| *SURVIVE-VT 2022* | *Some concerns* | *Low* | *Low* | *Low* | *High* | *High* |
| *VANISH2 2025* | *Low* | *Low* | *Low* | *Low* | *High* | *High* |
| *MANTRA-VT 2025* | *Low* | *Some concerns* | *Low* | *Low* | *High* | *High* |

**Table S2.** Summary of the risk-of-bias assessment for the included randomized controlled trials using the RoB 2 tool for the adverse events outcome, across standard methodological domains. Green indicates low risk of bias; yellow indicates some concerns; and red indicates high risk of bias.

**Supplementary Figure 1. All-cause mortality risk ratio**

*
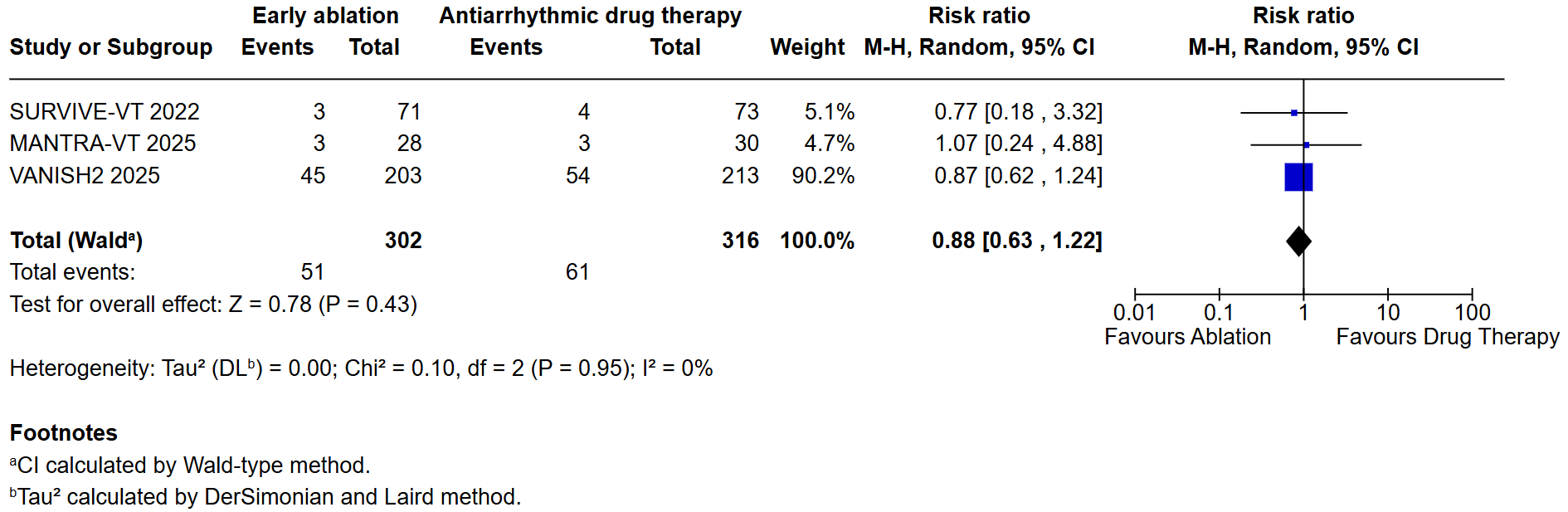
*

**Figure S1.** Forest plot comparing early catheter ablation versus antiarrhythmic drug therapy as first-line treatment for all-cause mortality in patients with ischemic cardiomyopathy, showing no significant difference (RR 0.88; 95% CI 0.63–1.22; I² = 0%).

**Supplementary Figure 2.** *Leave-one-out sensitivity analysis for adverse events.*


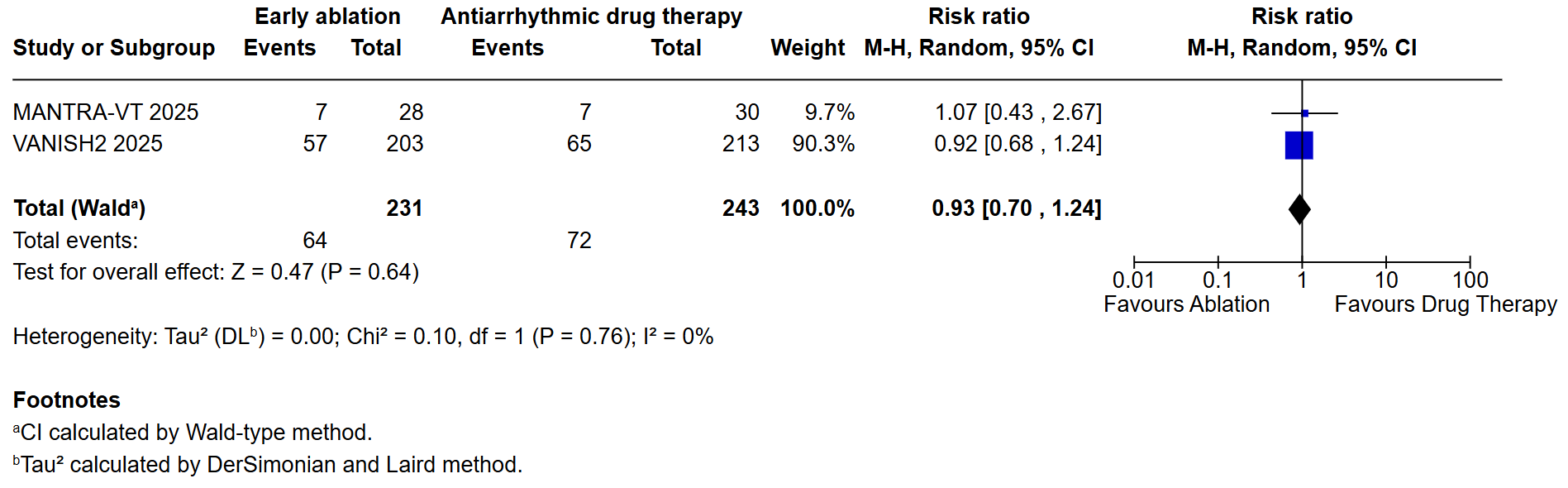


**Figure S2:** Leave-one-out sensitivity analysis for adverse events comparing early catheter ablation versus first-line antiarrhythmic drug therapy, showing no significant difference between strategies (RR 0.93; 95% CI 0.70–1.24; I² = 0%).

**Supplementary Figure 3.** *Leave-one-out sensitivity analysis for hospitalizations.*

**
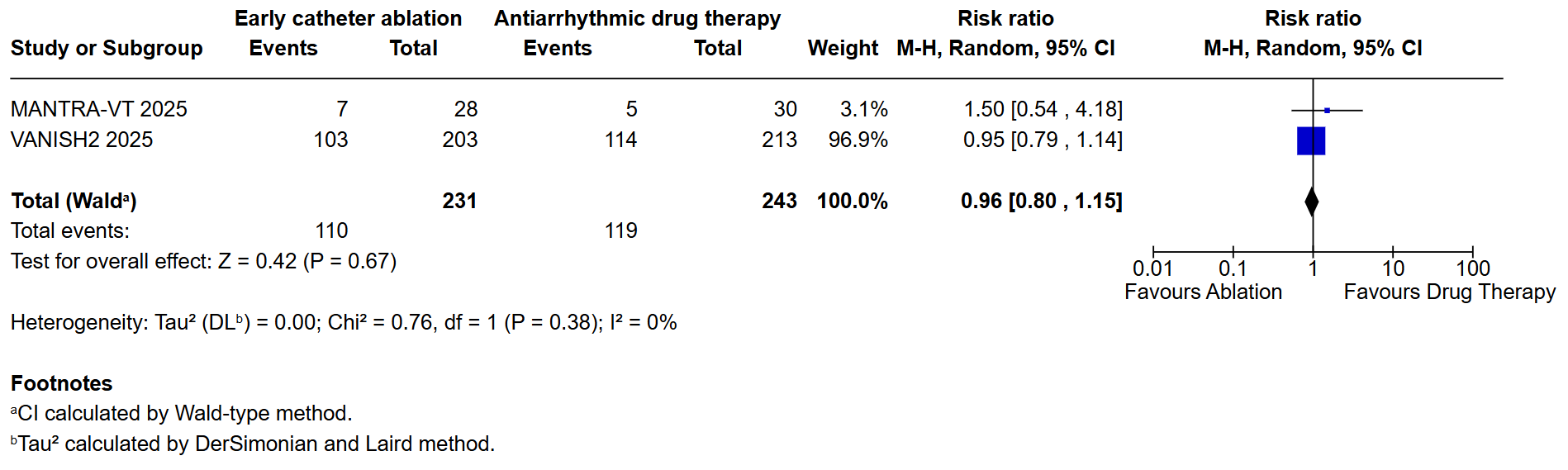
Figure S3:** Leave-one-out sensitivity analysis for hospitalizations comparing early catheter ablation versus first-line antiarrhythmic drug therapy, showing no significant difference between strategies (RR 0.96; 95% CI 0.80–1.15; I² = 0%).
